# Perceptions on artificial intelligence among anaesthesia and intensive care professionals: An international survey on attitudes, expectations and needs

**DOI:** 10.1007/s10877-026-01464-6

**Published:** 2026-07-04

**Authors:** Raphael Theilen, Tim Kramer, Martin Scharffenberg, Ian-Christopher Jung, Maria Zerlik, Marcelo Gama de Abreu, Saman Sepehr, Thea Koch, Brita Sedlmayr, Robert Huhle, Jakob Wittenstein

**Affiliations:** 1 Department of Anaesthesiology and Intensive Care Medicine, Pulmonary Engineering Group, Faculty of Medicine, University Hospital Carl Gustav Carus, TUD Dresden University of Technology, Dresden, Germany; 2 Institute for Medical Informatics and Biometry, Faculty of Medicine, University Hospital Carl Gustav Carus, TUD Dresden University of Technology, Fetscherstraße 74, 01307 Dresden, Germany; 3 Department of Intensive Care and Resuscitation, Anesthesiology Institute, Cleveland Clinic, Cleveland, OH USA; 4 Department of Outcomes Research, Anesthesiology Institute, Cleveland Clinic, Cleveland, OH USA; 5 European Society of Anaesthesiology and Intensive Care, Brussels, Belgium

**Keywords:** Artificial Intelligence, Decision Support System, Intensive Care Medicine, Anaesthesiology

## Abstract

*Background* Artificial intelligence (AI) can enhance diagnostics, treatment, and workflow efficiency. However, successful integration into clinical practice depends on users’ acceptance. *Objective* To investigate benefits, barriers, and challenges of AI applications among anaesthesia and intensive care professionals. *Design* International online survey. *Main outcome measures* The survey included items on familiarity and experiences with AI applications, perceived benefits, concerns, and demographic variables. Descriptive analyses, fisher exact tests, χ²-tests, odds ratios, and Spearman rank correlations were used to explore associations between responses and demographics. *Results* The survey was distributed by the European Society of Anaesthesiology and Intensive Care in 2023. A total of 510 respondents completed the entire survey, primarily from Europe (78%) and Asia (14.5%), and the majority were board-certified anaesthesiologists (86.3%). 86.5% of the respondents were aware of AI applications, but only 36.8% reported regular encounters. Familiarity was higher among males and intensive care specialists. 94.5% expressed interest in AI training, particularly younger and less experienced professionals. 94.7% expressed willingness to use AI applications, citing benefits such as improved decision-making (92.7%), complication anticipation (88.6%), and workload reduction (80.1%). Younger and female respondents were more optimistic about AI’s benefits. Key concerns included lack of explainability (68.4%), over-reliance on AI (80.8%), and medico-legal uncertainties (58.4%). Scepticism was attributed to insufficient validation studies and fears of inaccurate outputs, particularly among experienced clinicians. *Conclusions* This international survey shows cautious optimism among anaesthesia and intensive care professionals regarding AI applications. Adoption in clinical practice requires tailored training that accounts also for demographic-specific concerns, robust validation, and clear ethical and legal frameworks.

**Supplementary Information:**

The online version contains supplementary material available at https://doi.org/10.1007/s10877-026-01464-6.

## Introduction

 Recent years have shown a rapid expansion in artificial intelligence (AI)-based applications in healthcare. AI models can classify, integrate, and correlate large volumes of heterogeneous data by inferring complex data patterns through computer-generated algorithms. This enables the generation of patient-specific predictive models. In the field of intensive care medicine, these capabilities allow for the early prediction of hypovolemia severity and the subsequent need for massive transfusion protocols or damage-control surgery in trauma patients. This enables timely and targeted interventions. Furthermore, AI can identify and delineate distinct disease phenotypes in conditions such as sepsis and acute respiratory distress syndrome (ARDS), enabling more precise risk stratification and treatment selection. Finally, AI can generate dynamic, personalized risk trajectories for disease progression and mortality, supporting continuous clinical decision-making and opening new avenues for personalized, adaptive patient management [1–5]. Recognising its transformative potential, regulatory authorities worldwide have initiated measures to address associated legal, ethical, and practical challenges. For example, the European Union’s AI Act seeks to establish a robust and stringent regulatory framework for AI development in sensitive domains such as healthcare, safety and fundamental rights in which aspects such quality of data sets used, technical documentation and record-keeping, transparency, accuracy and cybersecurity are of essential importance [6]. Similar developments are underway globally, including China’s strategic policies to integrate AI across sectors, including healthcare [7].

Anaesthesia and intensive care medicine, disciplines characterized by the presence of large volumes of high-frequency data, present unique opportunities for the application of AI. AI-based decision-support systems (AI-DSS) offer the potential to enhance patient outcomes through early diagnosis, complication prediction, and optimal intervention strategies. However, successful implementation requires more than technological innovation and regulatory clarity. it may also depend on the trust, acceptance, and readiness of anaesthesia and intensive care professionals [8]. Clinicians are essential in integrating AI into routine care, making their perceptions central to its adoption.

Studies in fields such as radiology have explored clinicians’ perceptions of AI, highlighting both optimism and caution [9]. Within anaesthesia and intensive care, similar investigations remain a topic of high interest [9–12]. This survey aimed to analyse professional attitudes toward AI to facilitate its responsible and user-oriented development, to identify educational needs and to ensure its successful integration into clinical practice [13].

## Methods

### Ethical approval

This international voluntary survey was conducted on behalf of the IntelliLung Consortium and in accordance with the Declaration of Helsinki and its ethical principles [34]. Ethical approval (BO-EK-511112022) was granted by the institutional review board at the Medical Faculty of the Dresden University of Technology (TUD), Dresden, Germany (Chairperson: Prof. Bertold Renner) on 21 December 2022.

### Participants and survey distribution

The European Society of Anaesthesiology and Intensive Care (ESAIC) distributed an email with the survey link to 38,989 subscribers. The survey opened on January 23, 2023, with a reminder being sent again on March 6th to 30,000 subscribers. The same link was posted on the ESAIC Facebook page and on the IntelliLung project webpage from January 31 to July 31, 2023. Several national anaesthesiology societies were asked to share the survey. The survey was also promoted at the Euroanaesthesia 2023 conference (Glasgow, June 3–5, 2023) and at the same time it was promoted on ESAIC’s different social media platforms including LinkedIn, Twitter and Facebook. It was closed on July 31, 2023. ESAIC tracked the delivery and opening of emails. Additionally, the survey link remained accessible online. Following the invitation link, potential participants were informed about the voluntary nature of the survey and were asked to give consent, before being able to continue with the survey. After data cleaning, only fully completed questionnaires were analysed.

### Survey content

The questionnaire was developed based on a rapid literature review of existing surveys addressing AI applications [35–37]. Relevant items from previously published questionnaires were identified, reviewed for appropriateness, adapted specifically to the intensive care context, and compiled into an initial draft. Because the survey was exploratory and did not aim to establish a new psychometric scale or composite score, formal psychometric validation was beyond the scope of this study [38–40]. The questionnaires development was therefore limited to literature-informed item generation, expert review, and pretesting for clarity and face validity. The initial draft was pretested among a small group of respondents to evaluate clarity, redundancy, and time required for completion. Minor adjustments were made based on their feedback. The final questionnaire comprised 34 questions grouped into four sections: (1) Knowledge and experience with AI-based applications in clinical practice. (2) Expectations of AI-based DSS for clinical practice, including potential benefits and capabilities. (3) Concerns regarding AI-based DSS, including barriers to implementation, ethical and legal considerations, transparency of algorithms, and potential negative influences on clinical practice. (4) Personal, institutional, and professional demographics (e.g., age, gender, region, institution type, years of professional and ICU experience). Questions were primarily multiple-choice, often with a six-point rating scale ranging from “strongly disagree” to “strongly agree.” For descriptive presentation, varying degrees of agreement and disagreement (“somewhat agree/disagree,” “agree (disagree,” and “strongly agree/disagree”) were combined and reported as general agreement or disagreement respectively. Some questions allowed multiple selections or free-text answers. Adaptive branching was used, i.e. certain questions only appeared based on prior responses. The complete questionnaire is available in the online supplement (see Supplemental digital content 1). The survey was designed to be completed in in less than 15 min. Respondents were allowed to skip questions and use a back button to review answers. The internet protocol address -based restriction was applied to prevent multiple submissions. Incomplete responses (i.e., those not reaching demographic questions) were excluded since most demographics were asked at the end. No incentives were offered for participation. No representativeness weighting or propensity scores were applied.

### Statistical analysis

This was an exploratory, descriptive study. Thus, no previous sample size calculation was carried out. Associations between dichotomised Likert-scale responses and demographic variables were assessed using Fisher’s exact test for R×C contingency tables. To account for multiple testing across survey items and demographic variables, p-values from the omnibus tests were adjusted using the Benjamini-Hochberg false discovery rate procedure. For item-demographic combinations with a significant FDR-adjusted omnibus test, post-hoc pairwise comparisons between demographic subgroups were performed using χ² tests on the corresponding 2 × 2 contingency tables. These post-hoc p-values were adjusted using the Bonferroni method within each set of pairwise comparisons. Post-hoc analyses were used to identify which demographic subgroups contributed to significant omnibus associations. (SciPy 1.0) [41]. Unadjusted odds ratios (OR) and the 95%-confidence interval (CI) were calculated to assess the likelihood of general agreement (i.e., “somewhat agree,” “agree,” or “strongly agree”) among certain demographic groups. Spearman rank correlation measured associations between ordinal variables (e.g., age, years of experience) and response patterns. Significance was set at *P* < 0.05.

## Results

### Response rate and demographics

Of the initial 38,989 e-Blasts (survey invitations), 38,965 were successfully delivered, 16,212 recipients opened the email, and 454 clicked on the survey link. A reminder e-blast to 30,000 contacts led to 14,200 opens and 328 clicks. A total of 510 participants completed the survey. Most respondents (78%, *n* = 398) were from Europe, followed by Asia (14.5%, *n* = 74). The remainder were from other continents. The countries respondents were primarily working in are detailed in Table 1. The gender distribution was 57.1% male (*n* = 289), 40.8% female (*n* = 208), 2.4% (*n* = 12) preferred not to say, and one indicated “diverse.” Regarding the age distribution, 5.1% (*n* = 26) were < 30 years old, 31–40 years was the largest category (*n* = 157). 29.0% (*n* = 148) were 41–50, 22.7% (*n* = 116) were 51–60, and 10.4% (*n* = 53) were > 60 years old.

Most participants were board-certified in anaesthesiology (86.3%, *n* = 440). Additional certifications included intensive care (49.0%, *n* = 250) and critical emergency medicine (16.9%, *n* = 86). Multi-certifications were common: 30.8% (*n* = 157) had anaesthesiology and intensive care, and 11.0% (*n* = 56) held triple certification in anaesthesiology, intensive care, and emergency medicine. 37.5% (*n* = 191) were certified only in anaesthesiology.

Regarding the workplace, most respondents were affiliated with university hospitals (59.4%, *n* = 303), followed by community teaching hospitals (22.5%, *n* = 115). A minority worked at non-hospital or industrial-commercial sites. In terms of professional position, 43.1% (*n* = 220) were senior physicians, 31.4% (*n* = 160) were medical specialists, and 12.9% (*n* = 66) were residents. Clinic directors comprised 8.0% (*n* = 41), and 4.5% (*n* = 23) were non-physicians.

**Table 1:** Primary country of employment

Country	*n*	Country	*n*	Country	*n*	Country	*n*
Germany	118	Brazil	8	Serbia	4	Kuwait	2
Turkey	43	Poland	7	Indonesia	3	Namibia	1
Spain	22	Saudi Arabia	6	Singapore	3	Luxembourg	1
United Kingdom	20	China	6	Ireland	3	Bulgaria	1
Switzerland	16	Czech Republic	5	Jordan	2	Cyprus	1
Italy	16	Israel	5	Hungary	2	Moldova	1
Greece	14	Estonia	5	Slovenia	2	Kosovo	1
Croatia	14	Serbia	4	Finland	2	Tunisia	1
Belgium	14	Russia	4	Mexico	2	Other	1
Romania	14	Malta	4	Montenegro	2	Pakistan	1
Austria	14	Argentina	4	New Zealand	2	Vietnam	1
Netherlands	13	Australia	4	Colombia	2	Kenya	1
India	13	Denmark	3	Mexico	2	South Korea	1
Sweden	12	Slovakia	3	Montenegro	2	Qatar	1
Portugal	11	Bosnia and Herzegovina	3	New Zealand	2	Armenia	1
USA	9	Indonesia	3	Colombia	2	Georgia	1
France	9	Singapore	3	Canada	2	Syria	1
Norway	8	Ireland	3	Albania	2	Philippines	1

Country the respondents indicated to primarily work in, Count: Number of respondents to have indicated this country as their primary country of employment

### Knowledge and experience with AI

**Fig. 1:**
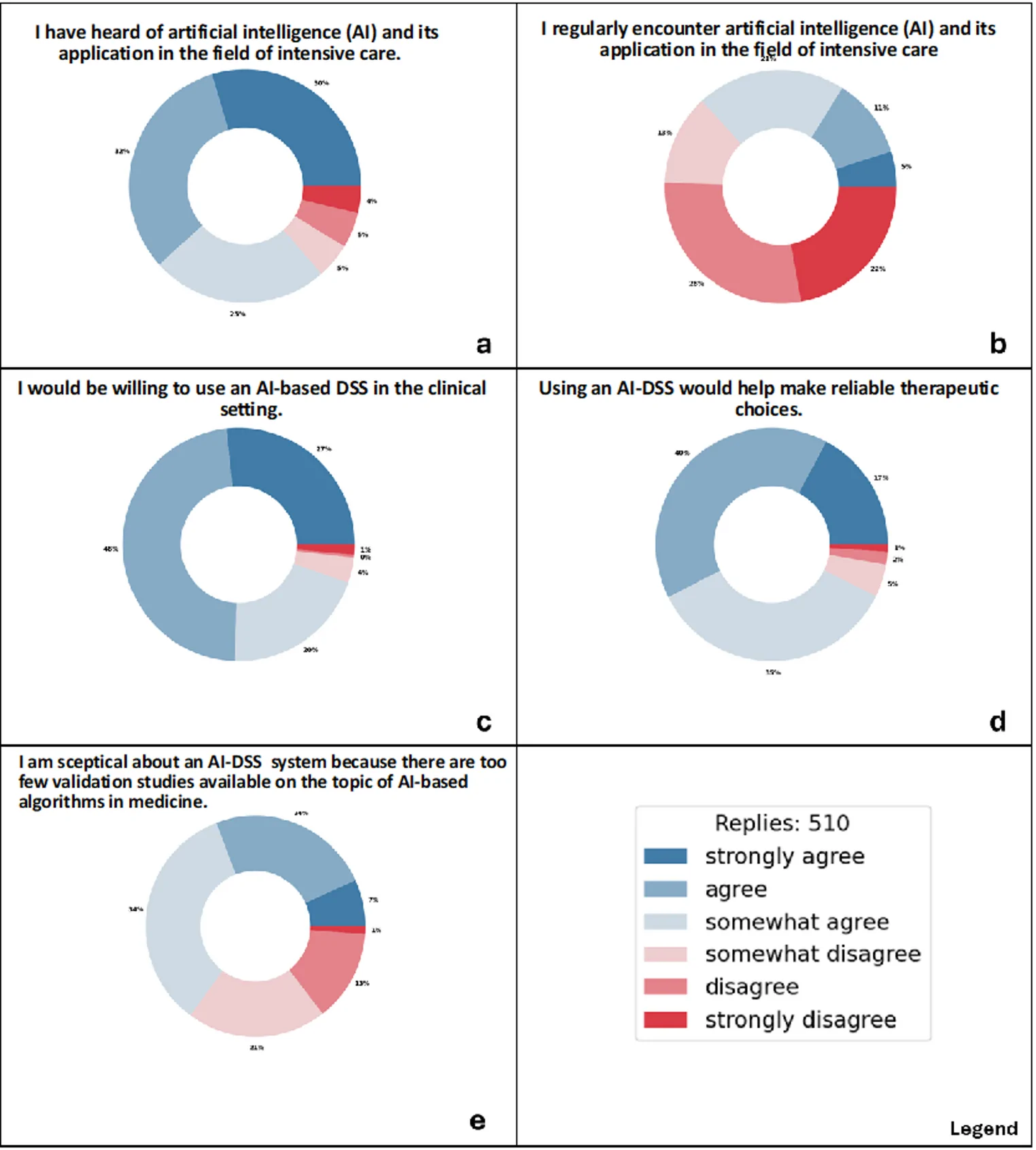
Respondents agreement to questionnaire items: **a**) I have heard of artificial intelligence (AI) and its application in the field of intensive care., **b**) I regularly encounter artificial intelligence (AI) and its application in the field of intensive care., **c**) I would be willing to use an AI-based DSS in the clinical setting., **d**) Using an AI-DSS would help make reliable therapeutic choices., **e**) I am sceptical about an AI-DSS system because there are too few validation studies available on the topic of AI-based algorithms in medicine

Overall, 86.5% (*n* = 441) agreed (at least somewhat) that they had heard of AI and its applications in intensive care (Fig. 1a). Male respondents showed a higher tendency to strongly agree (37.0%, *n* = 107) compared to female participants (19.7%, *n* = 41). Male respondents had an OR of 2.3 (CI 1.4–3.8, *P* = 0.002) for general agreement, while female respondents had an OR of 0.5 (CI 0.3–0.9, *P* = 0.010). More ICU experience correlated with a higher tendency to have encountered AI (Spearman’s ρ = 0.13, *P* = 0.002).

Despite widespread awareness, only 36.8% (*n* = 188) regularly encountered AI in their daily routine. (Fig. 1b). Although regular encounters among participants only numbered 188, more respondents report having met AI-applications at least once. These were closed-loop ventilation modes (68,2%, *n* = 348), assisted diagnostic aid systems (14,7%, *n* = 75) and decision support systems (13,3%, *n* = 68). The most frequently examples given by respondents were INTELLiVENT^®^-ASV^®^ and the Acumen™ Hypotension Prediction Index. Generative AI such as large language models like Chat-GPT by Open AI were only reported by two respondents in the context of this study.

A strong majority (92.4%, *n* = 471) agreed that AI’s importance in medicine had increased.

Participants who indicate previous knowledge or experience of AI-based applications in clinical practice perceive a significant increase in the importance of AI-based algorithms in medicine (OR = 3.7, CI 1.8–7.6, *P* < 0.001). The same respondents also indicate that they expect the implementation and the corresponding training of medical staff to be easier than respondents with no previous exposure to AI-based algorithms in medicine (OR 2.5, CI 1.3–4.5, *P* = 0.005). At the same time, respondents with more experience expect intensive care to benefit from AI-based decision support, e.g. helping with adjustment of mechanical ventilation strategies (OR = 2.7, CI 1.2–5.9, *P* = 0.019) and would be significantly more likely to use this type of decision support in their clinical routine (OR = 2.9, CI 1.2–6.9, *P* = 0.026). Even in the case of AI algorithms that are not logically reproducible, respondents who have been exposed to AI-based applications are less likely to reject their use (OR = 0.4, CI 0.2–0.8, *P* = 0.010). Similarly, this demographic group also sees fewer ethical problems (OR = 0.5, CI 0.3–0.9, *P* = 0.031). They also see a significantly lower risk regarding the chance of inaccurate results misleading their medical decision-making (OR = 0.4, CI 0.2–0.8, *P* = 0.007) or the dependence of modern medicine on digital applications and IT infrastructure (OR = 0.5, CI 0.3–0.96, *P* = 0.049).

### Interest in AI education and ease of adoption

Enthusiasm for AI education was high: 94.5% (*n* = 482) wanted to take an AI-related course. Respondents working primarily in the operating theatre were more likely (OR = 3.6, CI 1.7–7.8, *P* = 0.001) to express interest. Younger age and less experience correlated with higher interest (ρ=-0.17 to -0.18, all *P* < 0.010).

Learning new digital solutions was perceived as easy by 85.7% (*n* = 437). Younger cohorts and those with fewer years of experience were significantly more confident in their ability to adopt new digital solutions. For instance, respondents with < 5 years of clinical experience often strongly agreed (21.8%, *n* = 12), while those with > 30 years rarely did. Correlations between ease of learning and experience, as well as age, were negative (ρ=-0.21, *P* < 0.001 for experience and ρ=-0.24, *P* < 0.001 for age).

### Opportunities through AI-based applications

A substantial majority (94.7%, *n* = 483) were willing to use an AI-DSS in clinical settings (Fig. 1c). Approximately 74.5% agreed or strongly agreed with this statement, and only 1.8% disagreed or strongly disagreed. Differences emerged by workplace and speciality. Increasing age and experience correlated negatively with willingness to use an AI-DSS in clinical settings (ρ=-0.16, *P* < 0.001 for age; ρ=-0.11, *P* = 0.011 for clinical experience).

Approximately 79.4% (*n* = 405) would accept an AI system adjusting ventilator settings automatically (closed-loop ventilation). Additionally, 80.1% (*n* = 408) generally agreed that AI-DSS could reduce physician workload. Asian respondents showed higher odds of agreeing AI could reduce workload (OR = 5.1, CI 1.8–14.2, *P* = 0.002), while Europeans were less convinced (OR = 0.29, CI 0.1–0.6, *P* < 0.001). Similarly, older and more experienced physicians tended to be somewhat more sceptical.

Most respondents (92.7%, *n* = 473) believed AI-DSS would help to make reliable therapeutic choices (Fig. 1d). There were no significant differences in agreement across different clinical positions for this particular statement. A majority (60.0%, *n* = 306) agreed AI could improve intensive care in general, with female respondents more likely to believe so (OR = 3.1, CI 1.3–7.1, *P* = 0.010). Further, 88.6% (*n* = 452) felt that AI-DSS could help anticipate complications of mechanical ventilation, indicating a widely shared optimism about potential patient care improvements.

### Fears and concerns

Despite optimism, concerns remained. About 59.8% (*n* = 305) were sceptical due to fears of AI failing to deliver accurate results.

Previous disappointing experiences with digital tools influenced perceptions, with 77.3% (*n* = 394) expressing scepticism because past digital solutions had not met their expectations.

Lack of validation studies was another critical issue, as 64.9% (*n* = 331) indicated scepticism due to perceived insufficient clinical evidence (Fig. 1e).

A large majority (80.8%, *n* = 412) believed AI-DSS could increase physician dependency on technology.

Concerns about excessive reliance on digital infrastructure were reported by 70.4% (*n* = 359).

Moreover, 81.4% (*n* = 415) feared that AI recommendations might be accepted without reflection over time, and 68.4% (*n* = 349) would refuse to use an AI-DSS if its implementation rule was not transparently reproducible. Many (70.6%, *n* = 360) believed that the use of AI-DSS could impair conventional learning and understanding in future generations.

Medico-legal aspects also posed questions. Only 41.6% (*n* = 212) felt that responsibilities and liabilities were clear, while 31% disagreed or strongly disagreed with this notion. About 20.4% (*n* = 104) somewhat agreed and 15.1% (*n* = 77) somewhat disagreed. Ethical problems were perceived by 56.9% (*n* = 290).

## Discussion

This international survey among anaesthesia and intensive care professionals reveals a generally positive attitude toward AI-based applications. Most respondents recognized AI’s growing importance, expressed interest in AI training, and anticipated benefits such as improved reliability in decision-making, better anticipation of medical complications, and potential workload reductions. This survey features a large international sample with diverse professional backgrounds and its comprehensive coverage of attitudes toward AI-based decision support systems enhances the robustness and relevance of its findings. While caution is advised in generalizing the results broadly, the insights gained are valuable for understanding the current state of acceptance, expectations, and concerns related to AI in anaesthesiology and intensive care.

These findings align with previous surveys in radiology and other medical fields, which also reported cautious optimism and interest in AI adoption [9, 11–16]. However, some of these surveys had fewer respondents [9, 11], or only involved the perspective of healthcare professionals from the US, not Europe [12]. A similar observational study among 701 ESAIC members was conducted in 2021 [10], but more recent research suggests that perceptions of AI among anaesthesiologists have since evolved [17–19]. The strong willingness to consider AI-based applications such as AI-DSS suggests that, despite the technology’s relative novelty, anaesthesia and intensive care professionals are open to integrating such AI applications into clinical workflows. The limited familiarity in daily routine and number of actual encounters, as reported in this survey, is consistent with earlier findings as well [10, 20].

However, this positive attitude is tempered by notable reservations. Many respondents remain sceptical due to perceived “black-box” algorithms, lack of robust clinical validation, and uncertain ethical or legal frameworks. Algorithmic transparency and explainability emerged as key issues, echoing findings from other disciplines, where physicians prioritize their clinical judgment over AI-generated recommendations when the underlying rationale is unclear [20]. Legislative efforts, such as the EU AI Act, underline the importance of explainability in algorithmic decision-making and may help address these concerns [21]. However, at the time of this survey, medico-legal uncertainties appeared to be a critical barrier. The aforementioned efforts by global regulatory bodies to establish comprehensive legislation in this domain seem essential for the widespread acceptance of AI [6, 7, 22].

Our finding that demographic factors, such as gender or age significantly influenced perceptions of AI is in line with similar studies [17, 23]. Male respondents reported higher familiarity with AI compared to female respondents, who anticipated greater benefits in intensive care by for example leveraging AI to guide mechanical ventilation. This aligns with findings, that men tend to have a more positive attitude [24], greater self-assessed competence [25] and fewer concerns [26]. Younger and less experienced physicians demonstrated greater confidence in learning and utilizing AI applications, possibly due to higher digital literacy. Conversely, experienced clinicians, often having witnessed multiple technological shifts, were more cautious and less optimistic. These generational differences suggest that targeted educational initiatives may be essential to align varying levels of readiness and acceptance [10, 27].

Furthermore, prior experience with AI-based applications is associated with more favourable perceptions, with clinical benefits exceeding potential risks. This greater confidence aligns with previous findings [28] and is accompanied by increased willingness to adopt complex applications with not fully explainable algorithms at their core, as well as reduced concern about inaccurate outputs and the growing dependence of modern medicine on digital technologies.

Also consistent with previous studies [29], geographic differences influenced perceptions of AI. Asian respondents reported greater optimism regarding workload reduction This difference likely reflects variations in healthcare systems, regulatory frameworks [6, 7], and AI exposure, underscoring the importance of region-specific strategies to facilitate AI adoption.

A prominent concern among respondents was the potential erosion of clinical skills if future generations become overly reliant on AI-based systems, which is in line with previous findings [30, 31]. To mitigate this, AI should be framed as a supplementary, rather than substitutive, tool, reinforcing its role in enhancing and not replacing clinical decision-making. Educational programs integrating AI literacy with core clinical reasoning can help preserve fundamental competencies while promoting trust and confidence in these applications [32, 33].

This survey has several limitations. The findings of this study reflect the participants perspective on this topic rather than actual adoption readiness into clinical practice. The categorisations of general agreement/disagreement as detailed in Chap. 2.3 was used for interpretability and should not mistakenly be interpreted as uniform strong endorsement. Although the formal inferential analyses were restricted to selected demographic-item comparisons and adjusted for multiplicity, the survey remains exploratory in nature. Thus, subgroup findings should be interpreted as hypothesis-generating rather than confirmatory. Nurses and respiratory therapists were underrepresented, limiting the generalizability of their views. As this study was primarily aimed at healthcare personnel with duties in the ICU or operating theatre, opinions of healthcare providers not working there might not be properly represented in this study. Moreover, the findings of this study rather reflect the participants perspective on this topic rather than adoption readiness. Additionally, the majority of respondents were from Europe as the ESAIC subscribers are also mostly from Europe, and experienced professionals were overrepresented, potentially affecting the results. The voluntary nature of the survey may have introduced selection bias, attracting participants with pre-existing interest in AI. Furthermore, as this questionnaire mostly refers to AI-DSS or AI in general, the applicability of its findings to different types of AI-based applications in the clinical field (e.g. a peri-operative risk assessment tool or a monitoring aid) may be limited. Because the analyses were exploratory and involved multiple related tests, we needed to reduce the risk of false positive findings (type I error). This was done via the Benjamini-Hochberg procedure. Following the principle that multiplicity adjustments should be performed within logically related families of hypotheses, Benjamini-Hochberg correction was applied separately for each demographic variable across the survey items. Each demographic variable constituted a distinct family of hypotheses because the corresponding tests addressed different underlying constructs and were interpreted independently. This approach increases the likelihood of Type II errors (lower power) in exchange for stronger control of false positives. Effect sizes were reported with 95% confidence intervals and odds ratios, with non-significant findings treated as inconclusive rather than as evidence of no association. Lastly, this survey was conducted in the first half of 2023, meaning that the findings of this study should be applied with caution to the latest developments in AI-based applications in anaesthesia and intensive care.

## Conclusions

This international survey revealed cautious optimism among anaesthesia and intensive care professionals regarding the integration of AI into clinical practice. While respondents anticipated significant improvements in patient care, decision-making reliability, and efficiency, concerns about ethics, transparency, and legal clarity remain barriers to adoption. Tailored education, transparent AI design, rigorous validation, and the establishment of clear guidelines and regulatory frameworks are essential to address these challenges.

## Supplementary Information

Below is the link to the electronic supplementary material.

Supplementary Material 1

## Data Availability

The datasets used and/or analysed during the current study are available from the corresponding author upon reasonable request.

## Abbreviations

AI: Artificial Intelligence
AI-DSS: Artificial intelligence–based decision support system
CI: Confidence Interval
EU: European Union
ESAIC: European Society of Anaesthesiology and Intensive Care
ICU: Intensive care unit
OR: Odds ratio
