# Supplementary material for: Perceptions on artificial intelligence among anaesthesia and intensive care professionals: An international survey on attitudes, expectations and needs
Source: J Clin Monit Comput. 2026 Jul 4;40(5):1189–97. doi: 10.1007/s10877-026-01464-6 (PMC13562144; doi:10.1007/s10877-026-01464-6)
Supplement: Supplementary file 1 [file 10877_2026_1464_MOESM1_ESM.docx]

**Perceptions on Artificial Intelligence among Anaesthesia and Intensive Care Professionals: An international survey on attitudes, expectations, and needs**

Raphael Theilen^1#^, Tim Kramer^1#^, Martin Scharffenberg^1^, Ian-Christopher Jung^2^, Maria Zerlik^2^, Marcelo Gama de Abreu^1,3,4^, Saman Sepehr^5^, Thea Koch^1^, Brita Sedlmayr^2^, Robert Huhle^1^, Jakob Wittenstein^1^ for the IntelliLung Consortium

^1^ Department of Anaesthesiology and Intensive Care Medicine, Pulmonary Engineering Group, Faculty of Medicine and University Hospital Carl Gustav Carus, TUD Dresden University of Technology, Dresden, Germany;

^2^ Institute for Medical Informatics and Biometry, Faculty of Medicine and University Hospital Carl Gustav Carus, TUD Dresden University of Technology, Fetscherstraße 74, 01307 Dresden, Germany;

^3^ Department of Intensive Care and Resuscitation, Anesthesiology Institute, Cleveland Clinic, Cleveland, Ohio, USA;

^4^ Department of Outcomes Research, Anesthesiology Institute, Cleveland Clinic, Cleveland, Ohio, USA.

^5^ European Society of Anaesthesiology and Intensive Care, Brussels, Belgium

\# RaTh and TiKr share first authorship

**Corresponding author:** Dr. Jakob Wittenstein, MD, Department of Anaesthesiology and Intensive Care Medicine, Pulmonary Engineering Group, University Hospital Carl Gustav Carus, Technische Universität Dresden, Fetscherstrasse 74,01307 Dresden, Germany, Email address: [jakob](mailto:jakob).wittenstein@ukdd.de

# Online Supplement

# Members of the IntelliLung Consortium

| IntelliLung Consortium Member | Affiliation |
| --- | --- |
| Prof. Dr. med. Thea Koch | TECHNISCHE UNIVERSITAET DRESDEN |
| Dr. med. Jakob Wittenstein | > Professur für Anästhesiologie |
| Dr. med. Martin Scharffenberg | > Professur für Anästhesiologie |
| Dr. Robert Huhle | > Professur für Anästhesiologie |
| Tim Kramer | > Professur für Anästhesiologie |
| Raphael Theilen | > Professur für Anästhesiologie |
| Dr. Xina Grählert | > Koordinierungszentrum für Klinische Studien Dresden |
| Barbara Djawid | > Koordinierungszentrum für Klinische Studien Dresden |
| Julia Kalinka | > Koordinierungszentrum für Klinische Studien Dresden |
| Konstanty Szuldrzynski | Central Clinical Hospital of the Ministry of Interior and Administration in Warsaw |
| Agnieszka Pawlak | Central Clinical Hospital of the Ministry of Interior and Administration in Warsaw |
| Dr. Milosz Jankowski | Central Clinical Hospital of the Ministry of Interior and Administration in Warsaw |
| Valentina JANEV | Institut Mihajlo Pupin |
| Nikola Tomasevic | Institut Mihajlo Pupin |
| Lazar Berbakov | Institut Mihajlo Pupin |
| Dea Pujić | Institut Mihajlo Pupin |
| Milos Nenadovic | Institut Mihajlo Pupin |
| Rebekka Post | Was Hab' Ich? Gemeinnutzige Gmbh |
| Paula Hartmann | Was Hab' Ich? Gemeinnutzige Gmbh |
| (Aija) Marie Ladda | Was Hab' Ich? Gemeinnutzige Gmbh |
| Marijke de Cock | Demcon Macawi Respiratory Systems Bv |
| Koen Smit | Demcon Macawi Respiratory Systems Bv |
| Sytske Klomp | Demcon Macawi Respiratory Systems Bv |
| Ben van den Elshout | Demcon Macawi Respiratory Systems Bv |
| Patrick Kleuskens | Demcon Macawi Respiratory Systems Bv |
| Catherine Weynants | European Society Of Anaesthesiology And Intensive Care Aisbl |
| Sylvia Dameen | European Society Of Anaesthesiology And Intensive Care Aisbl |
| Ilse Decraen | European Society Of Anaesthesiology And Intensive Care Aisbl |
| Julia Dowell | European Society Of Anaesthesiology And Intensive Care Aisbl |
| Sarah De Coster | European Society Of Anaesthesiology And Intensive Care Aisbl |
| Saman Sepehr | European Society Of Anaesthesiology And Intensive Care Aisbl |
| Xavier Garcia | Better Care SL |
| Bernat Sales | Better Care SL |
| Jaume Montanya | Better Care SL |
| Enric Llopis | Better Care SL |
| Rudys Magrans | Better Care SL |
| Arnau Moral | Better Care SL |
| Sahar Vahdati | Institut für angewandte Informatik e.V. |
| Jason Li | Institut für angewandte Informatik e.V. |
| Farhad Safaei | Institut für angewandte Informatik e.V. |
| Nikhil Ostwal | Institut für angewandte Informatik e.V. |
| Jens LEHMANN | Institut für angewandte Informatik e.V. |
| Michael Martin | Institut für angewandte Informatik e.V. |
| Sabine Gruender-Fahrer | Institut für angewandte Informatik e.V. |
| Sandra Fuchs | Institut für angewandte Informatik e.V. |
| Roman Liesner | Institut für angewandte Informatik e.V. |
| Muhammad Hamza Yousuf | Institut für angewandte Informatik e.V. |
| Lorenzo Ball | University of Genoa |
| Prof. Marcelo Gama de Abreu | Cleveland Clinic Foundation |
| Catalina Dussan | Cleveland Clinic Foundation |
| Lluis BLANCH | Parc Tauli University Hospital |
| Leonardo Sarlabous Uranga | Parc Tauli University Hospital |
| Anna AGUILAR | Parc Tauli University Hospital |
| Fernando Suarez Sipmann | Hospital Universitario de La Princesa |
| Alfonso Canabal Berlanga | Hospital Universitario de La Princesa |
| Isabel Magaña Bru | Hospital Universitario de La Princesa |
| Miguel Ángel Armengol de la Hoz | Fundacion Publica Andaluza Progreso y Salud |
| Alvaro Ritore | Fundacion Publica Andaluza Progreso y Salud |
| Esther GUIRADO | Fundacion Publica Andaluza Progreso y Salud |
| Martin Faltys | InselSpital-Stiftung |

# Survey Content

# Statistical Results with adjusted P according Bonferroni–Holm

Demographic: Demographic being tested for agreement, Question Item: Question Item being tested for agreement, P-Value (Fisher exact test): P-Value according to Fisher exact-Test, Adjusted P-Value (Benjamini-Hochberg procedure): P-Value adjusted according to the Benjamini-Hochberg procedure.

| **Question item** | **Demographic** | **P-Value (Fisher exact test)** | **Adjusted P-Value (Benjamini-Hochberg procedure)** |
| --- | --- | --- | --- |
| Using an AI-DSS would help reduce the workload of physicians and staff. | Continent | 0,000407 | 0,008546991 |
| I would be interested in taking a course on AI-based applications in the field of medicine and intensive care. | Primary work environment | 0,001066999 | 0,022406978 |
| When applying an AI-DSS, the responsibilities are clear to me and I see no medico-legal problems. | Difficulties understanding english | 0,001217999 | 0,025577974 |
| I have heard of artificial intelligence (AI) and its application in the field of intensive care. | Gender | 0,002989997 | 0,042643957 |
| According to my personal experiences, new digital solutions in the clinical setting often did not meet expectations. | Country | 0,004321996 | 0,064028936 |
| Using an Al-based decision support system (DSS) that provides settings for mechanical ventilation would improve intensive care. | Gender | 0,005223995 | 0,042643957 |
| I foresee that the implementation of AI-based treatment suggestions would impair conventional learning and understanding of future generations of physicians. | Gender | 0,006091994 | 0,042643957 |
| When applying an AI-DSS, the responsibilities are clear to me and I see no medico-legal problems. | Country | 0,006097994 | 0,064028936 |
| I regularly encounter artificial intelligence (AI) and its application in the field of intensive care | Difficulties understanding english | 0,006703993 | 0,07039193 |
| I think using an AI-DSS would pose ethical problems. | Primary specialisation | 0,014505985 | 0,304625695 |
| Using an AI-DSS would help reduce the workload of physicians and staff. | Gender | 0,017283983 | 0,090740909 |
| According to my personal experiences, new digital solutions in the clinical setting often did not meet expectations. | Continent | 0,018365982 | 0,192842807 |
| I would be willing to use an AI-based DSS in the clinical setting. | Primary work environment | 0,020584979 | 0,216142284 |
| I am sceptical about an AI-DSS, because I fear the system might fail to deliver accurate results and could mislead my work with inaccurate suggestions. | Gender | 0,023167977 | 0,097305503 |
| Learning to use new digital solutions and applying them in a clinical setting is easy for me. | Age | 0,027677972 | 0,437715162 |
| Increasing dependency of treatments on digital solutions and IT infrastructure is a risk. | Country | 0,028011972 | 0,196083804 |
| I regularly encounter artificial intelligence (AI) and its application in the field of intensive care | Continent | 0,031976968 | 0,197594052 |
| Learning to use new digital solutions and applying them in a clinical setting is easy for me. | Clinical experience | 0,034088966 | 0,663893336 |
| Using an AI-DSS would help anticipate complications of ventilation. | Gender | 0,034784965 | 0,121747378 |
| I think using an AI-DSS would pose ethical problems. | Continent | 0,037636962 | 0,197594052 |
| I regularly encounter artificial intelligence (AI) and its application in the field of intensive care | ICU experience | 0,038430962 | 0,597774902 |
| When applying an AI-DSS, the responsibilities are clear to me and I see no medico-legal problems. | Primary specialisation | 0,042877957 | 0,45021855 |
| I think using an AI-DSS would pose ethical problems. | Age | 0,044903955 | 0,437715162 |
| Increasing dependency of treatments on digital solutions and IT infrastructure is a risk. | Difficulties understanding english | 0,054195946 | 0,379371621 |
| Using an Al-based decision support system (DSS) that provides settings for mechanical ventilation would improve intensive care. | ICU experience | 0,056930943 | 0,597774902 |
| I do not want to use an AI-DSS if the algorithm is not logically reproducible. | Primary work setting | 0,07010293 | 0,42030198 |
| I would be willing to use an AI-based DSS in the clinical setting. | Gender | 0,078326922 | 0,232226168 |
| I regularly encounter artificial intelligence (AI) and its application in the field of intensive care | Position at work | 0,081085919 | 0,55013435 |
| Using an Al-based decision support system (DSS) that provides settings for mechanical ventilation would improve intensive care. | Position at work | 0,082888917 | 0,55013435 |
| I think the use of an AI-DSS could lead to an increased dependency of physicians on technology. | Gender | 0,092388908 | 0,232226168 |
| Increasing dependency of treatments on digital solutions and IT infrastructure is a risk. | Clinical experience | 0,093466907 | 0,663893336 |
| I foresee that the implementation of AI-based treatment suggestions would impair conventional learning and understanding of future generations of physicians. | Primary work setting | 0,095121905 | 0,42030198 |
| I think the use of an AI-DSS could lead to an increased dependency of physicians on technology. | Position at work | 0,098230902 | 0,55013435 |
| I have heard of artificial intelligence (AI) and its application in the field of intensive care. | Age | 0,098421902 | 0,437715162 |
| I foresee that recommendations given by Al-DSS will increasingly be adapted without reflection. | Age | 0,103897896 | 0,437715162 |
| Using an Al-based decision support system (DSS) that provides settings for mechanical ventilation would improve intensive care. | Age | 0,104217896 | 0,437715162 |
| I am sceptical about an AI-DSS  system because there are too few validation studies available on the topic of AI-based algorithms in medicine. | Gender | 0,108008892 | 0,232226168 |
| The importance of AI-based algorithms in medicine has increased over the past years. | Gender | 0,110583889 | 0,232226168 |
| I am sceptical about an AI-DSS  system because there are too few validation studies available on the topic of AI-based algorithms in medicine. | Continent | 0,12033288 | 0,407324593 |
| Using an AI-DSS would help make reliable therapeutic choices. | Gender | 0,121960878 | 0,232834404 |
| I foresee that the implementation of AI-based treatment suggestions would impair conventional learning and understanding of future generations of physicians. | Difficulties understanding english | 0,122401878 | 0,53009947 |
| I am sceptical about an AI-DSS, because I fear the system might fail to deliver accurate results and could mislead my work with inaccurate suggestions. | Continent | 0,122653877 | 0,407324593 |
| According to my personal experiences, new digital solutions in the clinical setting often did not meet expectations. | Age | 0,126585873 | 0,443050557 |
| Using an AI-DSS would help make reliable therapeutic choices. | Primary work setting | 0,126936873 | 0,42030198 |
| I think using an AI-DSS would pose ethical problems. | Primary work setting | 0,128268872 | 0,42030198 |
| According to my personal experiences, new digital solutions in the clinical setting often did not meet expectations. | Clinical experience | 0,132279868 | 0,663893336 |
| I have heard of artificial intelligence (AI) and its application in the field of intensive care. | Primary work setting | 0,132443868 | 0,42030198 |
| When applying an AI-DSS, the responsibilities are clear to me and I see no medico-legal problems. | Continent | 0,135774864 | 0,407324593 |
|  | Position at work | 0,141772858 | 0,55013435 |
| Using an Al-based decision support system (DSS) that provides settings for mechanical ventilation would improve intensive care. | Primary work setting | 0,145831854 | 0,42030198 |
| When applying an AI-DSS, the responsibilities are clear to me and I see no medico-legal problems. | ICU experience | 0,150608849 | 0,76010124 |
| I am sceptical about an AI-DSS  system because there are too few validation studies available on the topic of AI-based algorithms in medicine. | Position at work | 0,150922849 | 0,55013435 |
| Using an AI-DSS would help make reliable therapeutic choices. | Primary work environment | 0,151246849 | 0,848207818 |
| I regularly encounter artificial intelligence (AI) and its application in the field of intensive care | Clinical experience | 0,155721844 | 0,663893336 |
| I think the use of an AI-DSS could lead to an increased dependency of physicians on technology. | Difficulties understanding english | 0,159068841 | 0,53009947 |
| When applying an AI-DSS, the responsibilities are clear to me and I see no medico-legal problems. | Primary work setting | 0,163592836 | 0,42030198 |
| I would be interested in taking a course on AI-based applications in the field of medicine and intensive care. | Primary work setting | 0,166117834 | 0,42030198 |
| Learning to use new digital solutions and applying them in a clinical setting is easy for me. | Difficulties understanding english | 0,171798828 | 0,53009947 |
| I foresee that the implementation of AI-based treatment suggestions would impair conventional learning and understanding of future generations of physicians. | Country | 0,172389828 | 0,71027929 |
| I am sceptical about an AI-DSS, because I fear the system might fail to deliver accurate results and could mislead my work with inaccurate suggestions. | Age | 0,173794826 | 0,47008033 |
| The importance of AI-based algorithms in medicine has increased over the past years. | Primary work environment | 0,175069825 | 0,848207818 |
| Using an Al-based decision support system (DSS) that provides settings for mechanical ventilation would improve intensive care. | Difficulties understanding english | 0,176699823 | 0,53009947 |
| I would be willing to use an AI-based DSS in the clinical setting. | Primary work setting | 0,181121819 | 0,42030198 |
| I foresee that the implementation of AI-based treatment suggestions would impair conventional learning and understanding of future generations of physicians. | Age | 0,184626815 | 0,47008033 |
| I regularly encounter artificial intelligence (AI) and its application in the field of intensive care | Primary specialisation | 0,187828812 | 0,964011036 |
| I would be interested in taking a course on AI-based applications in the field of medicine and intensive care. | Clinical experience | 0,198019802 | 0,663893336 |
| Learning to use new digital solutions and applying them in a clinical setting is easy for me. | Primary work setting | 0,2001438 | 0,42030198 |
|  | Continent | 0,205770794 | 0,509348763 |
| According to my personal experiences, new digital solutions in the clinical setting often did not meet expectations. | Position at work | 0,207275793 | 0,55013435 |
| Using an AI-DSS would help reduce the workload of physicians and staff. | Age | 0,207518792 | 0,47008033 |
| According to my personal experiences, new digital solutions in the clinical setting often did not meet expectations. | ICU experience | 0,21034679 | 0,76010124 |
| Using an AI-DSS would help reduce the workload of physicians and staff. | Country | 0,213962786 | 0,71027929 |
| In general, I would accept an AI-system adjusting my ventilation-settings automatically in a closed-loop approach. | ICU experience | 0,215015785 | 0,76010124 |
| Increasing dependency of treatments on digital solutions and IT infrastructure is a risk. | ICU experience | 0,217171783 | 0,76010124 |
| I am sceptical about an AI-DSS, because I fear the system might fail to deliver accurate results and could mislead my work with inaccurate suggestions. | Primary work environment | 0,218437782 | 0,848207818 |
| Using an AI-DSS would help reduce the workload of physicians and staff. | Clinical experience | 0,218661781 | 0,663893336 |
| I am sceptical about an AI-DSS, because I fear the system might fail to deliver accurate results and could mislead my work with inaccurate suggestions. | Clinical experience | 0,221297779 | 0,663893336 |
| I regularly encounter artificial intelligence (AI) and its application in the field of intensive care | Age | 0,223847776 | 0,47008033 |
| I have heard of artificial intelligence (AI) and its application in the field of intensive care. | Position at work | 0,227364773 | 0,55013435 |
| Using an AI-DSS would help make reliable therapeutic choices. | Position at work | 0,228075772 | 0,55013435 |
| I am sceptical about an AI-DSS  system because there are too few validation studies available on the topic of AI-based algorithms in medicine. | Country | 0,236244764 | 0,71027929 |
| I regularly encounter artificial intelligence (AI) and its application in the field of intensive care | Country | 0,236759763 | 0,71027929 |
| Increasing dependency of treatments on digital solutions and IT infrastructure is a risk. | Continent | 0,237141763 | 0,509348763 |
| I do not want to use an AI-DSS if the algorithm is not logically reproducible. | Position at work | 0,252717747 | 0,55013435 |
| I think the use of an AI-DSS could lead to an increased dependency of physicians on technology. | Continent | 0,261616738 | 0,509348763 |
| I would be willing to use an AI-based DSS in the clinical setting. | Position at work | 0,261968738 | 0,55013435 |
| I do not want to use an AI-DSS if the algorithm is not logically reproducible. | Continent | 0,266801733 | 0,509348763 |
| According to my personal experiences, new digital solutions in the clinical setting often did not meet expectations. | Difficulties understanding english | 0,271981728 | 0,713952036 |
| I would be interested in taking a course on AI-based applications in the field of medicine and intensive care. | Gender | 0,275545724 | 0,473370219 |
| I am sceptical about an AI-DSS  system because there are too few validation studies available on the topic of AI-based algorithms in medicine. | Clinical experience | 0,284238716 | 0,7003353 |
| In general, I would accept an AI-system adjusting my ventilation-settings automatically in a closed-loop approach. | Gender | 0,293038707 | 0,473370219 |
| I foresee that recommendations given by Al-DSS will increasingly be adapted without reflection. | ICU experience | 0,299401701 | 0,898205102 |
|  | Clinical experience | 0,3001437 | 0,7003353 |
| Using an AI-DSS would help reduce the workload of physicians and staff. | Primary work setting | 0,306088694 | 0,581214669 |
| Using an Al-based decision support system (DSS) that provides settings for mechanical ventilation would improve intensive care. | Continent | 0,318439682 | 0,557269443 |
|  | Primary work environment | 0,322368678 | 0,848207818 |
| I think using an AI-DSS would pose ethical problems. | Gender | 0,323181677 | 0,484772515 |
| Using an AI-DSS would help make reliable therapeutic choices. | Difficulties understanding english | 0,32978567 | 0,767181733 |
| The importance of AI-based algorithms in medicine has increased over the past years. | Position at work | 0,33048467 | 0,599096588 |
| I foresee that recommendations given by Al-DSS will increasingly be adapted without reflection. | Country | 0,331288669 | 0,774768559 |
| In general, I would accept an AI-system adjusting my ventilation-settings automatically in a closed-loop approach. | Country | 0,332043668 | 0,774768559 |
| I am sceptical about an AI-DSS  system because there are too few validation studies available on the topic of AI-based algorithms in medicine. | Primary work setting | 0,332122668 | 0,581214669 |
| Using an Al-based decision support system (DSS) that provides settings for mechanical ventilation would improve intensive care. | Clinical experience | 0,335085665 | 0,703679896 |
| I think the use of an AI-DSS could lead to an increased dependency of physicians on technology. | Primary work environment | 0,342255658 | 0,848207818 |
| Using an AI-DSS would help make reliable therapeutic choices. | ICU experience | 0,353295647 | 0,927401073 |
| I have heard of artificial intelligence (AI) and its application in the field of intensive care. | Primary work environment | 0,354091646 | 0,848207818 |
| Using an AI-DSS would help anticipate complications of ventilation. | Primary work environment | 0,363517636 | 0,848207818 |
| Using an AI-DSS would help reduce the workload of physicians and staff. | Difficulties understanding english | 0,365324635 | 0,767181733 |
| In general, I would accept an AI-system adjusting my ventilation-settings automatically in a closed-loop approach. | Age | 0,367638632 | 0,652528847 |
| Using an AI-DSS would help reduce the workload of physicians and staff. | Position at work | 0,369496631 | 0,599096588 |
| Using an AI-DSS would help anticipate complications of ventilation. | Age | 0,372873627 | 0,652528847 |
| According to my personal experiences, new digital solutions in the clinical setting often did not meet expectations. | Primary specialisation | 0,374364626 | 0,964011036 |
| Learning to use new digital solutions and applying them in a clinical setting is easy for me. | Primary specialisation | 0,4000176 | 0,964011036 |
|  | Position at work | 0,405247595 | 0,599096588 |
| I am sceptical about an AI-DSS, because I fear the system might fail to deliver accurate results and could mislead my work with inaccurate suggestions. | Primary specialisation | 0,414171586 | 0,964011036 |
| I think using an AI-DSS would pose ethical problems. | Position at work | 0,417231583 | 0,599096588 |
| I do not want to use an AI-DSS if the algorithm is not logically reproducible. | Gender | 0,41958958 | 0,587425413 |
| I would be interested in taking a course on AI-based applications in the field of medicine and intensive care. | Age | 0,425086575 | 0,686678313 |
| Learning to use new digital solutions and applying them in a clinical setting is easy for me. | Primary work environment | 0,425421575 | 0,881072119 |
| Using an AI-DSS would help reduce the workload of physicians and staff. | Primary specialisation | 0,426570573 | 0,964011036 |
| In general, I would accept an AI-system adjusting my ventilation-settings automatically in a closed-loop approach. | Primary specialisation | 0,427179573 | 0,964011036 |
| Increasing dependency of treatments on digital solutions and IT infrastructure is a risk. | Primary specialisation | 0,435418565 | 0,964011036 |
| In general, I would accept an AI-system adjusting my ventilation-settings automatically in a closed-loop approach. | Position at work | 0,437634562 | 0,599096588 |
| I think using an AI-DSS would pose ethical problems. | Country | 0,438481562 | 0,88639628 |
| When applying an AI-DSS, the responsibilities are clear to me and I see no medico-legal problems. | Gender | 0,447939552 | 0,587920662 |
| I am sceptical about an AI-DSS, because I fear the system might fail to deliver accurate results and could mislead my work with inaccurate suggestions. | Position at work | 0,456454544 | 0,599096588 |
| Using an AI-DSS would help anticipate complications of ventilation. | Difficulties understanding english | 0,462714537 | 0,883364117 |
| Using an AI-DSS would help make reliable therapeutic choices. | Primary specialisation | 0,471111529 | 0,964011036 |
| According to my personal experiences, new digital solutions in the clinical setting often did not meet expectations. | Primary work setting | 0,481016519 | 0,700742699 |
| Using an AI-DSS would help anticipate complications of ventilation. | Primary work setting | 0,482823517 | 0,700742699 |
| Learning to use new digital solutions and applying them in a clinical setting is easy for me. | ICU experience | 0,49011151 | 0,969037399 |
| In general, I would accept an AI-system adjusting my ventilation-settings automatically in a closed-loop approach. | Primary work setting | 0,500530499 | 0,700742699 |
| I would be interested in taking a course on AI-based applications in the field of medicine and intensive care. | ICU experience | 0,504334496 | 0,969037399 |
| I foresee that the implementation of AI-based treatment suggestions would impair conventional learning and understanding of future generations of physicians. | Continent | 0,506994493 | 0,818991104 |
| I have heard of artificial intelligence (AI) and its application in the field of intensive care. | Primary specialisation | 0,517004483 | 0,964011036 |
| I foresee that the implementation of AI-based treatment suggestions would impair conventional learning and understanding of future generations of physicians. | ICU experience | 0,51963048 | 0,969037399 |
| I am sceptical about an AI-DSS, because I fear the system might fail to deliver accurate results and could mislead my work with inaccurate suggestions. | Difficulties understanding english | 0,522155478 | 0,913772086 |
| I am sceptical about an AI-DSS  system because there are too few validation studies available on the topic of AI-based algorithms in medicine. | Age | 0,524248476 | 0,786372714 |
| In general, I would accept an AI-system adjusting my ventilation-settings automatically in a closed-loop approach. | Primary work environment | 0,525918474 | 0,881072119 |
| Learning to use new digital solutions and applying them in a clinical setting is easy for me. | Country | 0,530568469 | 0,88639628 |
| Using an AI-DSS would help anticipate complications of ventilation. | Country | 0,537185463 | 0,88639628 |
| I regularly encounter artificial intelligence (AI) and its application in the field of intensive care | Primary work setting | 0,537485463 | 0,70544967 |
| Increasing dependency of treatments on digital solutions and IT infrastructure is a risk. | Gender | 0,53957046 | 0,657308676 |
| I foresee that the implementation of AI-based treatment suggestions would impair conventional learning and understanding of future generations of physicians. | Position at work | 0,54973445 | 0,644377356 |
| I foresee that recommendations given by Al-DSS will increasingly be adapted without reflection. | Continent | 0,55043445 | 0,825651674 |
| I would be willing to use an AI-based DSS in the clinical setting. | Primary specialisation | 0,550863449 | 0,964011036 |
| I foresee that recommendations given by Al-DSS will increasingly be adapted without reflection. | Position at work | 0,552323448 | 0,644377356 |
| According to my personal experiences, new digital solutions in the clinical setting often did not meet expectations. | Gender | 0,563407437 | 0,657308676 |
| The importance of AI-based algorithms in medicine has increased over the past years. | Age | 0,567270433 | 0,794178606 |
| I regularly encounter artificial intelligence (AI) and its application in the field of intensive care | Primary work environment | 0,567867432 | 0,881072119 |
| I think the use of an AI-DSS could lead to an increased dependency of physicians on technology. | Primary work setting | 0,576940423 | 0,712691111 |
| I am sceptical about an AI-DSS  system because there are too few validation studies available on the topic of AI-based algorithms in medicine. | Primary work environment | 0,57950742 | 0,881072119 |
| I foresee that recommendations given by Al-DSS will increasingly be adapted without reflection. | Primary work environment | 0,587381413 | 0,881072119 |
| I have heard of artificial intelligence (AI) and its application in the field of intensive care. | Country | 0,59000441 | 0,88639628 |
| I am sceptical about an AI-DSS, because I fear the system might fail to deliver accurate results and could mislead my work with inaccurate suggestions. | ICU experience | 0,616503383 | 0,969037399 |
| Using an AI-DSS would help reduce the workload of physicians and staff. | ICU experience | 0,626622373 | 0,969037399 |
| I foresee that recommendations given by Al-DSS will increasingly be adapted without reflection. | Gender | 0,627390373 | 0,693431464 |
| In general, I would accept an AI-system adjusting my ventilation-settings automatically in a closed-loop approach. | Clinical experience | 0,641817358 | 0,987102013 |
| I do not want to use an AI-DSS if the algorithm is not logically reproducible. | Age | 0,642094358 | 0,817578594 |
| I would be interested in taking a course on AI-based applications in the field of medicine and intensive care. | Difficulties understanding english | 0,655447345 | 0,957932571 |
| I think the use of an AI-DSS could lead to an increased dependency of physicians on technology. | Age | 0,661849338 | 0,817578594 |
| I have heard of artificial intelligence (AI) and its application in the field of intensive care. | ICU experience | 0,664712335 | 0,969037399 |
| Increasing dependency of treatments on digital solutions and IT infrastructure is a risk. | Primary work setting | 0,667999332 | 0,779332554 |
| I foresee that the implementation of AI-based treatment suggestions would impair conventional learning and understanding of future generations of physicians. | Clinical experience | 0,668948331 | 0,987102013 |
| Using an AI-DSS would help anticipate complications of ventilation. | Clinical experience | 0,671421329 | 0,987102013 |
| I do not want to use an AI-DSS if the algorithm is not logically reproducible. | Country | 0,671683328 | 0,88639628 |
| I have heard of artificial intelligence (AI) and its application in the field of intensive care. | Continent | 0,678851321 | 0,839811327 |
| I think the use of an AI-DSS could lead to an increased dependency of physicians on technology. | Country | 0,685164315 | 0,88639628 |
| Using an AI-DSS would help anticipate complications of ventilation. | Continent | 0,688526311 | 0,839811327 |
| I think using an AI-DSS would pose ethical problems. | Difficulties understanding english | 0,689058311 | 0,957932571 |
| I am sceptical about an AI-DSS  system because there are too few validation studies available on the topic of AI-based algorithms in medicine. | Primary specialisation | 0,711753288 | 0,985911014 |
| I foresee that recommendations given by Al-DSS will increasingly be adapted without reflection. | Primary specialisation | 0,716241284 | 0,985911014 |
| In general, I would accept an AI-system adjusting my ventilation-settings automatically in a closed-loop approach. | Difficulties understanding english | 0,717712282 | 0,957932571 |
| I would be willing to use an AI-based DSS in the clinical setting. | Continent | 0,719223281 | 0,839811327 |
| In general, I would accept an AI-system adjusting my ventilation-settings automatically in a closed-loop approach. | Continent | 0,71983828 | 0,839811327 |
| I think the use of an AI-DSS could lead to an increased dependency of physicians on technology. | ICU experience | 0,721683278 | 0,969037399 |
| Increasing dependency of treatments on digital solutions and IT infrastructure is a risk. | Position at work | 0,723525276 | 0,794328406 |
| I would be willing to use an AI-based DSS in the clinical setting. | Difficulties understanding english | 0,735347265 | 0,957932571 |
| Using an Al-based decision support system (DSS) that provides settings for mechanical ventilation would improve intensive care. | Primary specialisation | 0,743617256 | 0,985911014 |
| I foresee that recommendations given by Al-DSS will increasingly be adapted without reflection. | Primary work setting | 0,747239253 | 0,825896016 |
| I would be willing to use an AI-based DSS in the clinical setting. | Country | 0,751480249 | 0,88639628 |
| Using an AI-DSS would help make reliable therapeutic choices. | Country | 0,753575246 | 0,88639628 |
|  | Age | 0,755896244 | 0,874349599 |
| I would be interested in taking a course on AI-based applications in the field of medicine and intensive care. | Position at work | 0,756503243 | 0,794328406 |
| I am sceptical about an AI-DSS, because I fear the system might fail to deliver accurate results and could mislead my work with inaccurate suggestions. | Country | 0,75976824 | 0,88639628 |
| I do not want to use an AI-DSS if the algorithm is not logically reproducible. | Clinical experience | 0,762362238 | 0,987102013 |
| I have heard of artificial intelligence (AI) and its application in the field of intensive care. | Difficulties understanding english | 0,775469225 | 0,957932571 |
| Increasing dependency of treatments on digital solutions and IT infrastructure is a risk. | Age | 0,791078209 | 0,874349599 |
| I regularly encounter artificial intelligence (AI) and its application in the field of intensive care | Gender | 0,795415205 | 0,835185965 |
| The importance of AI-based algorithms in medicine has increased over the past years. | Continent | 0,802991197 | 0,887516586 |
|  | Primary work setting | 0,803177197 | 0,837448163 |
| When applying an AI-DSS, the responsibilities are clear to me and I see no medico-legal problems. | Clinical experience | 0,815421185 | 0,987102013 |
| Increasing dependency of treatments on digital solutions and IT infrastructure is a risk. | Primary work environment | 0,816344184 | 0,991712008 |
| The importance of AI-based algorithms in medicine has increased over the past years. | Clinical experience | 0,819072181 | 0,987102013 |
| Using an AI-DSS would help anticipate complications of ventilation. | ICU experience | 0,824076176 | 0,969037399 |
| Using an AI-DSS would help make reliable therapeutic choices. | Clinical experience | 0,834726165 | 0,987102013 |
| I am sceptical about an AI-DSS, because I fear the system might fail to deliver accurate results and could mislead my work with inaccurate suggestions. | Primary work setting | 0,837448163 | 0,837448163 |
| Learning to use new digital solutions and applying them in a clinical setting is easy for me. | Gender | 0,843306157 | 0,843306157 |
| The importance of AI-based algorithms in medicine has increased over the past years. | Difficulties understanding english | 0,848268152 | 0,966881033 |
|  | ICU experience | 0,859486141 | 0,969037399 |
| When applying an AI-DSS, the responsibilities are clear to me and I see no medico-legal problems. | Primary work environment | 0,866680133 | 0,991712008 |
| I think using an AI-DSS would pose ethical problems. | ICU experience | 0,872906127 | 0,969037399 |
| I do not want to use an AI-DSS if the algorithm is not logically reproducible. | Difficulties understanding english | 0,875689124 | 0,966881033 |
| Using an AI-DSS would help make reliable therapeutic choices. | Continent | 0,876615123 | 0,92044588 |
| I am sceptical about an AI-DSS  system because there are too few validation studies available on the topic of AI-based algorithms in medicine. | ICU experience | 0,876748123 | 0,969037399 |
| I would be interested in taking a course on AI-based applications in the field of medicine and intensive care. | Country | 0,877706122 | 0,938893061 |
| I would be willing to use an AI-based DSS in the clinical setting. | Age | 0,87998312 | 0,881273119 |
| When applying an AI-DSS, the responsibilities are clear to me and I see no medico-legal problems. | Age | 0,881273119 | 0,881273119 |
| I do not want to use an AI-DSS if the algorithm is not logically reproducible. | Primary specialisation | 0,888673111 | 0,985911014 |
| Using an AI-DSS would help reduce the workload of physicians and staff. | Primary work environment | 0,9004401 | 0,991712008 |
| I do not want to use an AI-DSS if the algorithm is not logically reproducible. | ICU experience | 0,923511076 | 0,96968663 |
| I foresee that recommendations given by Al-DSS will increasingly be adapted without reflection. | Difficulties understanding english | 0,928542071 | 0,966881033 |
| The importance of AI-based algorithms in medicine has increased over the past years. | Country | 0,932836067 | 0,938893061 |
| I think using an AI-DSS would pose ethical problems. | Clinical experience | 0,934102066 | 0,987102013 |
| The importance of AI-based algorithms in medicine has increased over the past years. | Primary specialisation | 0,938123062 | 0,985911014 |
| I foresee that the implementation of AI-based treatment suggestions would impair conventional learning and understanding of future generations of physicians. | Primary specialisation | 0,938204062 | 0,985911014 |
| Using an Al-based decision support system (DSS) that provides settings for mechanical ventilation would improve intensive care. | Country | 0,938893061 | 0,938893061 |
| I have heard of artificial intelligence (AI) and its application in the field of intensive care. | Clinical experience | 0,938936061 | 0,987102013 |
| I would be interested in taking a course on AI-based applications in the field of medicine and intensive care. | Primary specialisation | 0,941259059 | 0,985911014 |
| I think the use of an AI-DSS could lead to an increased dependency of physicians on technology. | Primary specialisation | 0,942290058 | 0,985911014 |
| I foresee that the implementation of AI-based treatment suggestions would impair conventional learning and understanding of future generations of physicians. | Primary work environment | 0,945057055 | 0,991712008 |
| I would be willing to use an AI-based DSS in the clinical setting. | Clinical experience | 0,956082044 | 0,987102013 |
| I think using an AI-DSS would pose ethical problems. | Primary work environment | 0,964259036 | 0,991712008 |
| I am sceptical about an AI-DSS  system because there are too few validation studies available on the topic of AI-based algorithms in medicine. | Difficulties understanding english | 0,966881033 | 0,966881033 |
| Using an AI-DSS would help anticipate complications of ventilation. | Position at work | 0,96958503 | 0,96958503 |
| According to my personal experiences, new digital solutions in the clinical setting often did not meet expectations. | Primary work environment | 0,96959303 | 0,991712008 |
| Using an AI-DSS would help anticipate complications of ventilation. | Primary specialisation | 0,985911014 | 0,985911014 |
| I think the use of an AI-DSS could lead to an increased dependency of physicians on technology. | Clinical experience | 0,987102013 | 0,987102013 |
| I do not want to use an AI-DSS if the algorithm is not logically reproducible. | Primary work environment | 0,991712008 | 0,991712008 |
| I would be interested in taking a course on AI-based applications in the field of medicine and intensive care. | Continent | 1 | 1 |
| I would be willing to use an AI-based DSS in the clinical setting. | ICU experience | 1 | 1 |
